# Economic evaluation of liraglutide versus sitagliptin in the treatment of type 2 diabetes mellitus inadequately controlled with metformin

**DOI:** 10.3389/fcdhc.2026.1906364

**Published:** 2026-08-07

**Authors:** Fengkun Yang, Xiaohui Zheng, Wenqi Li, Zhenying Zhao

**Affiliations:** Department of Pharmacy, Tianjin Union Medical Center, The First Affiliated Hospital of Nankai University, Tianjin, China

**Keywords:** economic evaluation, liraglutide, Markov model, sitagliptin, type 2 diabetes mellitus

## Abstract

**Objective:**

To assess the long-term economic value of liraglutide compared with sitagliptin among patients with type 2 diabetes mellitus (T2DM) who fail to achieve adequate glycemic control with metformin monotherapy.

**Methods:**

A Markov model based on China’s health system was constructed. Using data from the 1860-LIRA-DPP-4 study, long-term costs, and utilities over a 30-year horizon were simulated for Chinese T2DM patients receiving liraglutide or sitagliptin in combination with metformin. The incremental cost-effectiveness ratio (ICER) was estimated against a willingness-to-pay (WTP) threshold of three times the 2025 per capita gross domestic product (GDP) in China. One-way sensitivity analysis, probabilistic sensitivity analysis, and scenario analysis were performed to validate the robustness of the base-case results.

**Results:**

Thirty-year disease simulation showed that, compared with sitagliptin plus metformin, liraglutide plus metformin yielded an ICER of 267,985.96 RMB/QALY, which was below the predefined WTP threshold, indicating that it is cost-effective. One-way sensitivity analysis revealed that costs in the complication-free state for the intervention group, utility values for uncomplicated diabetes, and utility values for complicated diabetes had the greatest impacts on the ICER. Probabilistic sensitivity analysis showed that liraglutide plus metformin was cost effective for 63.6% of the iterations. Scenario analysis indicated that the economic advantage of liraglutide plus metformin became more pronounced as the price of liraglutide decreased.

**Conclusion:**

Within the Chinese healthcare setting, for patients with T2DM inadequately controlled on metformin monotherapy, liraglutide plus metformin has a 63.6% probability of being cost-effective at the pre-specified WTP threshold, suggesting that the combination regimen is likely to be cost-effective.

## 1 Introduction

Diabetes mellitus is a disorder characterized by persistently elevated blood glucose levels due to insufficient insulin secretion, leading to systemic metabolic disorders and a series of clinical complications (1). Type 2 diabetes mellitus represents more than 90% of all diagnosed cases of diabetes in China (2). Diabetic complications severely impair patients’ quality of life and impose a substantial economic burden on society (3). As reported in a previous study (4), the global prevalence of diabetes among adults aged 20–79 years was 10.5% in 2021, affecting an estimated 536.6 million individuals. This prevalence is projected to increase to 12.2% by 2045, corresponding to approximately 783.2 million people. Worldwide diabetes-related healthcare spending totaled USD 966 billion in 2021 and is anticipated to rise to USD 1054 billion by 2045, marking a 9.1% increase. In 2021, total diabetes-related expenditures in China reached $165.3 billion USD, with a per capita expenditure of approximately $1,173.5 USD, which is equivalent to approximately 8,026.35 CNY per capita (5). In this context, conducting economic evaluations of antidiabetic medications to identify safe, effective, economical, and appropriate therapeutic regimens for patients is critical.

Metformin has long been the first-line pharmacotherapy for type 2 diabetes mellitus (T2DM). Both the Guidelines for the Prevention and Treatment of Diabetes in China (2024 Edition) issued by the Chinese Diabetes Society (6) and the Standards of Medical Care in Diabetes (2024) released by the American Diabetes Association (7) recommend adding a glucagon-like peptide-1 receptor agonist (GLP-1 RA) or a dipeptidyl peptidase-4 inhibitor (DPP-4i) when glycemic control remains inadequate with metformin alone. Liraglutide is the world’s first long-acting once-daily subcutaneous GLP-1 receptor agonist and was approved in China for weight management in 2023. It can reduce HbA1c levels by up to 1.5% in patients with T2DM, lower systolic blood pressure, and reduce the risk of cardiovascular diseases (8). Sitagliptin is the first DPP-4i approved for monotherapy or combination therapy in adults with T2DM. It significantly affects glycemic control, improves the insulinogenic index, suppresses glucagon secretion, reduces glycated hemoglobin, and preserves pancreatic β-cell function, with a low incidence of adverse reactions (9). However, the relatively high cost of GLP-1 RAs and DPP-4is has substantially increased medical expenses for patients with T2DM and placed additional pressure on healthcare insurance funds. Therefore, it is necessary to compare the long-term cost-effectiveness of liraglutide and sitagliptin in real-world Chinese clinical practice to support clinicians in selecting more economical and effective regimens and optimizing the allocation of scarce healthcare resources. Given this background on China’s healthcare system, the present study used data from the 1860-LIRA-DPP-4 head-to-head clinical trial comparing the efficacy and safety of liraglutide versus sitagliptin in patients with T2DM inadequately controlled with metformin (10) to perform a cost-utility analysis to assess the long-term economic value of liraglutide versus sitagliptin in this patient population in China.

## 2 Materials and methods

### 2.1 Target population and data source

The target population of this study was adult patients with T2DM with inadequate glycemic control on metformin, which is consistent with the patients enrolled in the 1860-LIRA-DPP-4 study, a randomized, open-label, controlled clinical trial (10). This multicenter, international clinical study assessed the efficacy and safety of liraglutide or sitagliptin as add-on therapies to metformin in patients with T2DM who exhibited inadequate glycemic control on metformin monotherapy. The primary efficacy endpoints were changes in the glycated hemoglobin (HbA1c) level and body weight from baseline at week 52. The primary safety endpoints comprised treatment-emergent adverse events and symptomatic hypoglycemic episodes. We have presented our data in accordance with the updated 2022 CHEERS reporting checklist.

Inclusion criteria: (1) Age ≥ 18 years; (2) Confirmed diagnosis of T2DM with an HbA1c level of 7.5%–10.0%; and (3) Treatment with metformin for at least 3 months prior to study enrollment and having received outpatient endocrine care. Exclusion criteria: (1) Use of glucose-lowering agents other than metformin within 3 months before study enrollment; (2) Contraindications to liraglutide or sitagliptin; (3) Impaired hepatic or renal function; (4) Concomitant use of any medications other than metformin that may affect blood glucose levels; (5) Presence of malignant cancer; or (6) Clinically significant cardiovascular disease.

### 2.2 Interventions

The control regimen in the 1860-LIRA-DPP-4 study was sitagliptin (100 mg once daily orally) plus metformin at a dose of ≥1500 mg/day or the maximum tolerated dose. The investigational regimen was liraglutide (1.2 mg once daily via subcutaneous injection) plus metformin at a dose of ≥1500 mg/day or the maximum tolerated dose. The first 26 weeks of the trial constituted the main phase, followed by a 26-week extension phase (10).

Since the mean daily dose of metformin was not specified in the 1860-LIRA-DPP-4 study, based on the evidence base of this study, namely, the Standards of Medical Care in Diabetes (2024) issued by the American Diabetes Association, the optimal effective dose of metformin is 2000 mg/day (7), which is also the defined daily dose of metformin. Therefore, in this study, the investigational group received liraglutide (1.2 mg/day) plus metformin (2000 mg/day), and the control group received sitagliptin (100 mg/day) plus metformin (2000 mg/day), with the administration of each drug consistent with that in the 1860-LIRA-DPP-4 study.

### 2.3 Construction of the pharmacoeconomic model

**Figure 1:**
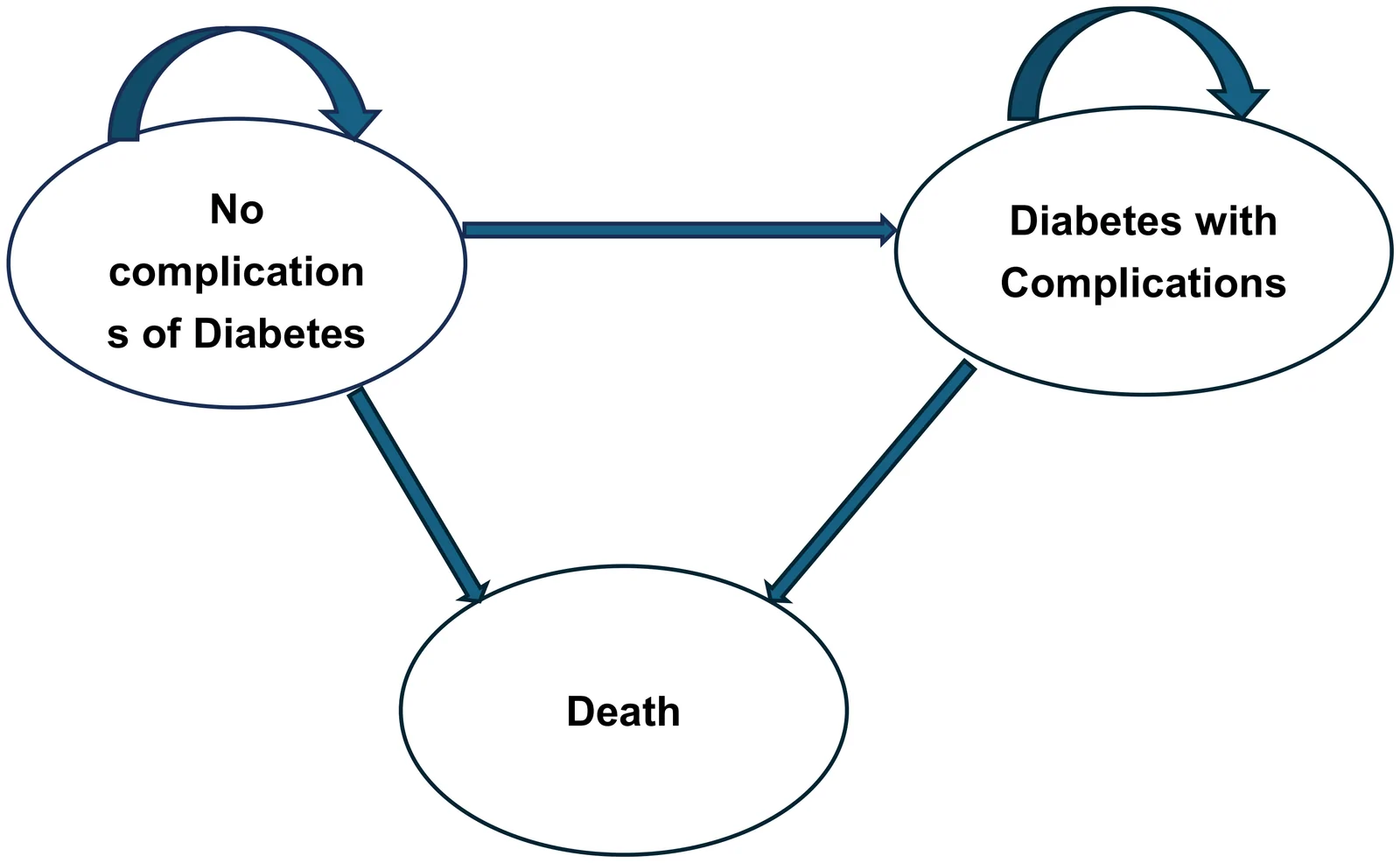
Markov model.

At present, a variety of pharmacoeconomic models for T2DM have been developed worldwide, such as the IHE model and the MICADO model. However, most of these models are based on the Markov model (11). The Markov model is the dominant model for the pharmacoeconomic evaluation of T2DM. Therefore, TreePro 2011 software was used in this study. According to the natural history of type 2 diabetes, the disease course is generally divided into three stages: uncomplicated type 2 diabetes, type 2 diabetes with complications, and the death state, with death occurring in an absorbing state. These three states were shown in **Figure 1**. Complications of type 2 diabetes include diabetic cerebrovascular disease, diabetic cardiovascular disease, diabetic nephropathy, and diabetic retinopathy, all of which are categorized under the state of type 2 diabetes with complications (12). Moreover, this study assumed the following: (1) no additional costs are incurred in the death state; (2) patient adherence to treatment is 100%; (3) the state of diabetes with complications includes both macrovascular and microvascular complications to simplify the model. (4) Patients were assumed to remain on liraglutide or sitagliptin combined with metformin throughout the 30-year simulation horizon, without modeling treatment intensification (e.g., addition of other antihyperglycemic agents).In addition, in accordance with the progression characteristics of type 2 diabetes, the model should reflect the long-term differences in prognosis between the interventions as much as possible. According to the National Health Commission of China, the average life expectancy in China in 2025 was 79.25 years, and the mean age of patients in the 1860-LIRA-DPP-4 study was 55 years. Therefore, in this study, a cycle length of 1 year was set, with the model running for 30 cycles, and half-cycle correction was applied. According to the 2020 Chinese Guidelines for Pharmacoeconomic Evaluation, a 5% discount rate was used for both costs and health outcomes in this model (13).

### 2.4 Model parameters

#### 2.4.1 Costs

This study was performed from the perspective of China’s healthcare system, and only direct medical costs associated with the treatment regimens were included in the analysis. Since the incidence of adverse reactions was low and the reactions are generally mild in both treatment groups and no special intervention was required (14, 15), the costs of managing adverse reactions were not considered. For patients with T2DM, glycemic control is achieved mainly through pharmacotherapy based on lifestyle interventions, and regular outpatient follow-up is needed. Complications may develop if glycemic control is inadequate. Therefore, the direct medical costs of the two treatment regimens included drug costs and basic treatment costs (background costs of examinations and laboratory tests, as well as costs of complication management).

Drug prices in this study were derived from the medical insurance catalog of Tianjin. The unit prices were as follows: metformin hydrochloride tablets (0.5 g×20 tablets), 0.9575 CNY per tablet; sitagliptin tablets (100 mg×7 tablets), 5.296 CNY per tablet; liraglutide injection (3 mL:18 mg), 315.27 CNY per pen. Basic treatment costs were calculated based on references (16, 17) and converted to 2025 prices using the Chinese Consumer Price Index. The background costs of examinations and laboratory tests and the costs of diabetic complications were 1590.81 CNY and 20005.87 CNY, respectively. For the liraglutide plus metformin group, the cost for uncomplicated diabetes was 10660.33 CNY, and the cost for complicated diabetes was 30666.20 CNY. For the sitagliptin plus metformin group, the cost for uncomplicated diabetes was 4921.70 CNY, and the cost for complicated diabetes was 24927.57 CNY. The annual costs associated with drug treatment are summarized in **Table 1**.

**Table 1:** Annual cost of drug treatment.

Drug	Specification	Unit price (CNY)	Manufacturer	Annual cost (CNY/year)
Metformin	0.5 g × 20 tablets	19.15	Merck Pharmaceutical Co., Ltd.	1397.95
Liraglutide	3 mL:18 mg	315.27	Novo Nordisk Pharmaceutical Co., Ltd.	7671.57
Sitagliptin	100 mg × 7 tablets	37.07	MSD Pharmaceutical Co., Ltd.	1932.94

#### 2.4.2 Transition probabilities

Given that the clinical trial adopted in this study did not provide detailed statistics on diabetic complications, the annual transition probabilities of T2DM progressing to various complications could not be calculated directly from the trial data. Therefore, the transition probabilities for each health state under the two treatment regimens were derived based on the results from the United Kingdom Prospective Diabetes Study (UKPDS) (18). The study population consisted of patients receiving metformin monotherapy, from which the baseline annual incidence rates of macrovascular complications, microvascular complications, and death after metformin treatment could be determined. According to the results from the DCCT trial (19), when HbA1c was maintained between 7% and 11%, a 10% relative improvement in the HbA1c concentration was associated with a 40% reduction in complication risk. Based on this relationship, the relative risk reductions of complications in patients from the two treatment groups were estimated. In the 1860-LIRA-DPP-4 study (10), patients in the liraglutide group achieved a mean HbA1c reduction of 1.29% from baseline (8.4%), representing a 15.36% relative improvement in HbA1c, corresponding to a 61.44% reduction in complication risk. Patients in the sitagliptin group achieved a mean HbA1c reduction of 0.88% from baseline (8.5%), representing a 10.35% relative improvement in HbA1c, corresponding to a 41.4% reduction in complication risk. The annual incidence rates of complications and mortality for patients receiving the two treatment regimens are presented in **Table 2**. The incidence rates were converted to probabilities using the following formula: p(t)=1−e−rt (where p=annual transition probability, r=incidence rate, and t=time interval for the incidence rate) (20). Annual transition probabilities between health states for the two treatment regimens are presented in **Table 3**.

**Table 2:** Annual incidence of complications and mortality in patients in the two treatment regimens.

Treatment regimen	Macrovascular complication incidence	Microvascular complication incidence	Non-vascular mortality incidence	Overall mortality incidence
Intervention group	0.0081	0.00258	0.00262	0.0052
Control group	0.01231	0.00393	0.00398	0.00791

**Table 3:** Annual transition probabilities among various health states in patients.

Transition state	Intervention group	Control group
Uncomplicated diabetes → Uncomplicated diabetes	0.9868	0.9799
Uncomplicated diabetes → Complicated diabetes	0.0106	0.0161
Uncomplicated diabetes → Death	0.0026	0.0040
Complicated diabetes → Complicated diabetes	0.9974	0.9961
Complicated diabetes → Death	0.0026	0.0039

#### 2.4.3 Utility values and discount rate

Given that authoritative utility data for patients with diabetes with complications are currently lacking in China, the utility values in this study were derived from the study by Redekop (21). Specifically, the utility value for uncomplicated diabetes was 0.81, that for diabetes with complications was 0.69, and that for death was 0. The discount rate was set at 5%, as recommended by the pharmacoeconomic guidelines (13).

### 2.5 Analytical methods

#### 2.5.1 Cohort simulation

A cost-utility analysis was performed to compare the economic value of the various treatment regimens. The primary outcome measure was the incremental cost-effectiveness ratio (ICER). According to pharmacoeconomic guidelines (13), 1 to 3 times China’s per capita gross domestic product (GDP) is commonly used as the willingness-to-pay (WTP) threshold per quality-adjusted life year (QALY). An ICER that is below 1 times per capita GDP indicates that the additional cost is fully worthwhile, between 1 and 3 times per capita GDP indicates that the additional cost is acceptable, and above 3 times per capita GDP indicates that the additional cost is not worthwhile. Based on data from the National Bureau of Statistics, China’s per capita GDP in 2025 was 99,665 CNY. Therefore, the WTP threshold in this study was set at 3 times China’s per capita GDP, equivalent to 298,995 CNY.

#### 2.5.2 Sensitivity analysis

One-way sensitivity analysis was conducted to examine the effects of changes in key model parameters on the results. The parameter variation ranges were set as 0-8% for the discount rate (13), ± 10% for health utility values and ±20% for baseline cost values (22). Findings of the one-way sensitivity analysis are presented in a tornado diagram.

Probabilistic sensitivity analysis was also performed using 1,000 Monte Carlo simulations to evaluate the simultaneous effects of multiple parameters varying according to their respective statistical distributions. The standard deviation for the probabilistic parameters was calculated as (upper limit-lower limit)/(2×1.96) (22). The findings are illustrated by a cost-effectiveness scatter plot and a cost-effectiveness acceptability curve. The input values of the key parameters in the probabilistic sensitivity analysis are shown in **Table 4**.

**Table 4:** Input values for key parameters in the probabilistic sensitivity analysis.

Parameter	Baseline	Lower limit	Upper limit	Standard deviation	Distribution type
Discount rate/%	0.05	0	0.08	0.02	Normal
Treatment cost (CNY/year)					
Intervention group, diabetes without complications	10660.33	8528.264	12792.396	1087.7888	Gamma
Intervention group, diabetes with complications	30666.20	24532.960	36799.440	3129.2041	Gamma
Control group, diabetes without complications	4921.70	3937.360	5906.040	502.2143	Gamma
Control group, diabetes with complications	24927.57	19942.056	29913.084	2543.6296	Gamma
Health utility value					
Diabetes without complications	0.81	0.729	0.891	0.0413	Beta
Diabetes with complications	0.69	0.621	0.759	0.0352	Beta

#### 2.5.3 Scenario analysis

In this study, we assumed that the price of liraglutide would be reduced by 20%, 40%, and 60%, respectively, based on its price in the Medical Insurance Catalog. The cost-utility of the two treatment regimens after 30 cycles was evaluated and compared.

## 3 Results

### 3.1 Results of the base-case analysis

After 30 cycles of model simulation, 67.12% of the patients in the intervention group had no complications, 25.37% developed complications, and 7.51% died. In the control group, 54.38% of patients had no complications, 34.34% developed complications of varying severity, and 11.28% died. Patients treated with liraglutide combined with metformin had lower probabilities of complications and death.

The cost-utility analysis showed that compared with the control regimen, the intervention regimen increased costs by 74,609.97 CNY, with an additional utility gain of 0.278 QALY. The calculated ICER was 267,985.96 CNY/QALY, which was lower than the WTP threshold set in this study (1 to 3 times the per capita GDP), indicating that the additional cost was economically acceptable. Therefore, the intervention regimen was cost-effective. The results are presented in **Table 5**.

**Table 5:** Results of the base-case analysis.

Treatment regimen	Cumulative cost/CNY	Incremental cost/CNY	Cumulative utility/QALY	Incremental utility/QALY	ICER/(CNY/QALY)
Intervention group	196802.05	74609.97	12.188	0.278	267985.96
Control group	122192.08	—	11.910	—	—

### 3.2 Results of the sensitivity analysis

#### 3.2.1 Results of probabilistic sensitivity analysis

**Figure 2:**
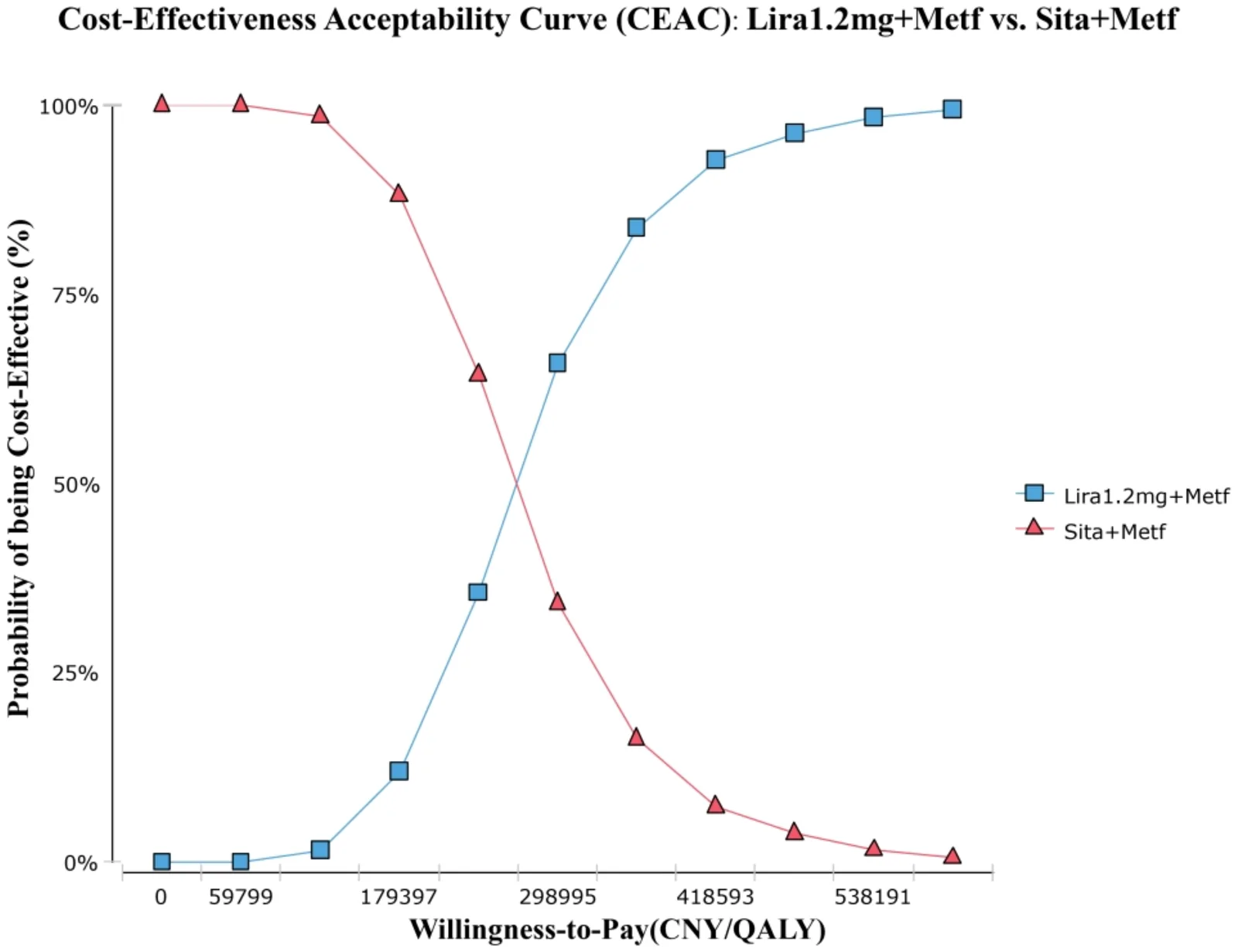
Cost-effectiveness acceptability curves.

**Figure 3:**
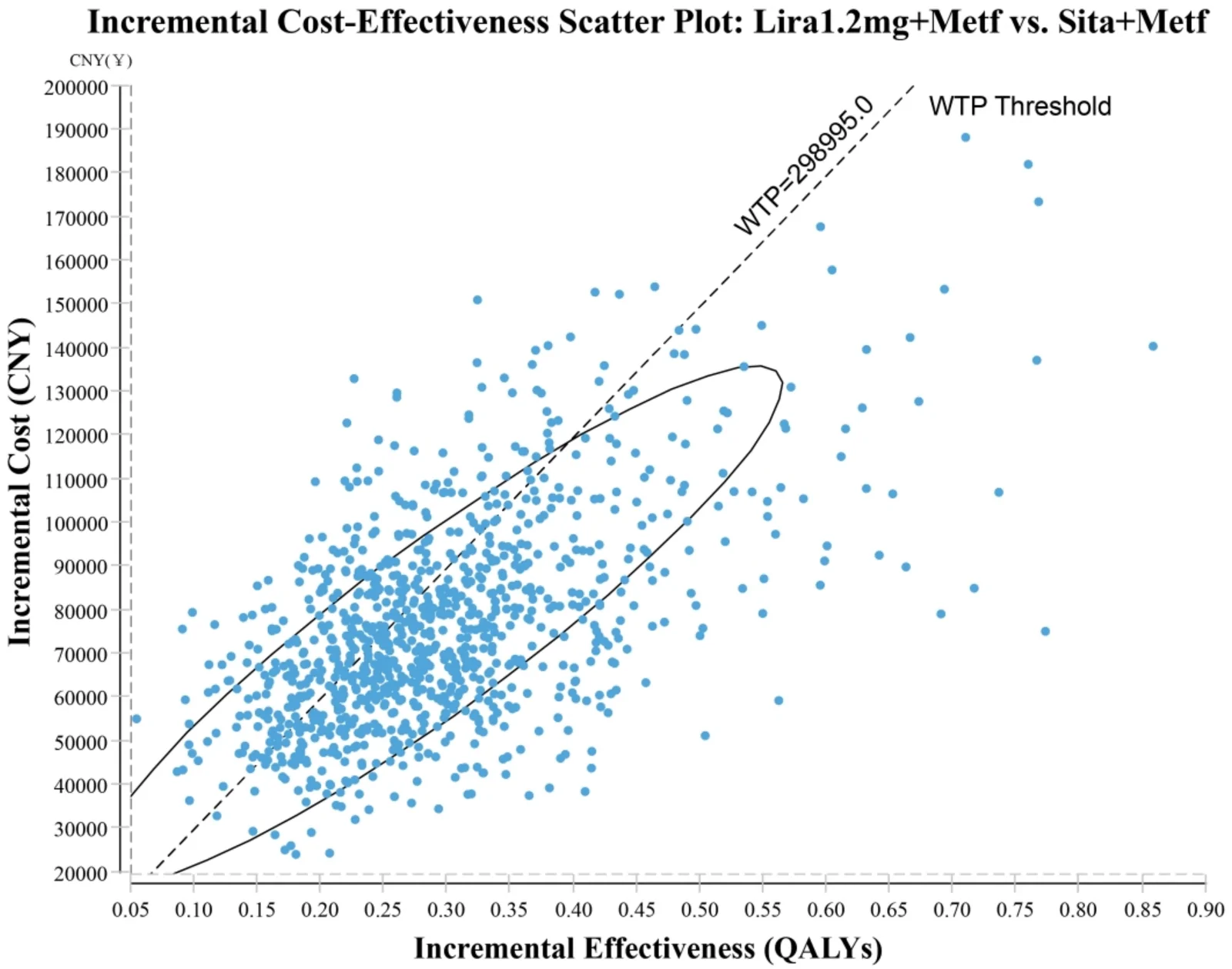
Cost-effectiveness scatter plot.

Monte Carlo sampling was performed with 1000 simulations to generate scatter plots comparing the intervention group versus the control group for the treatment of T2DM. The results are shown in **Figures 2**, **3**.

As shown in **Figure 2**, the probability that the experimental treatment is cost-effective increases with the willingness-to-pay (WTP) threshold.

**Figure 3** reveals that all ICER scatter plots fall within the first quadrant, suggesting stable results. At a WTP threshold of ¥298,995/QALY, the experimental regimen has a 63.6% probability of being cost-effective.

#### 3.2.2 Results of one-way sensitivity analysis

**Figure 4:**
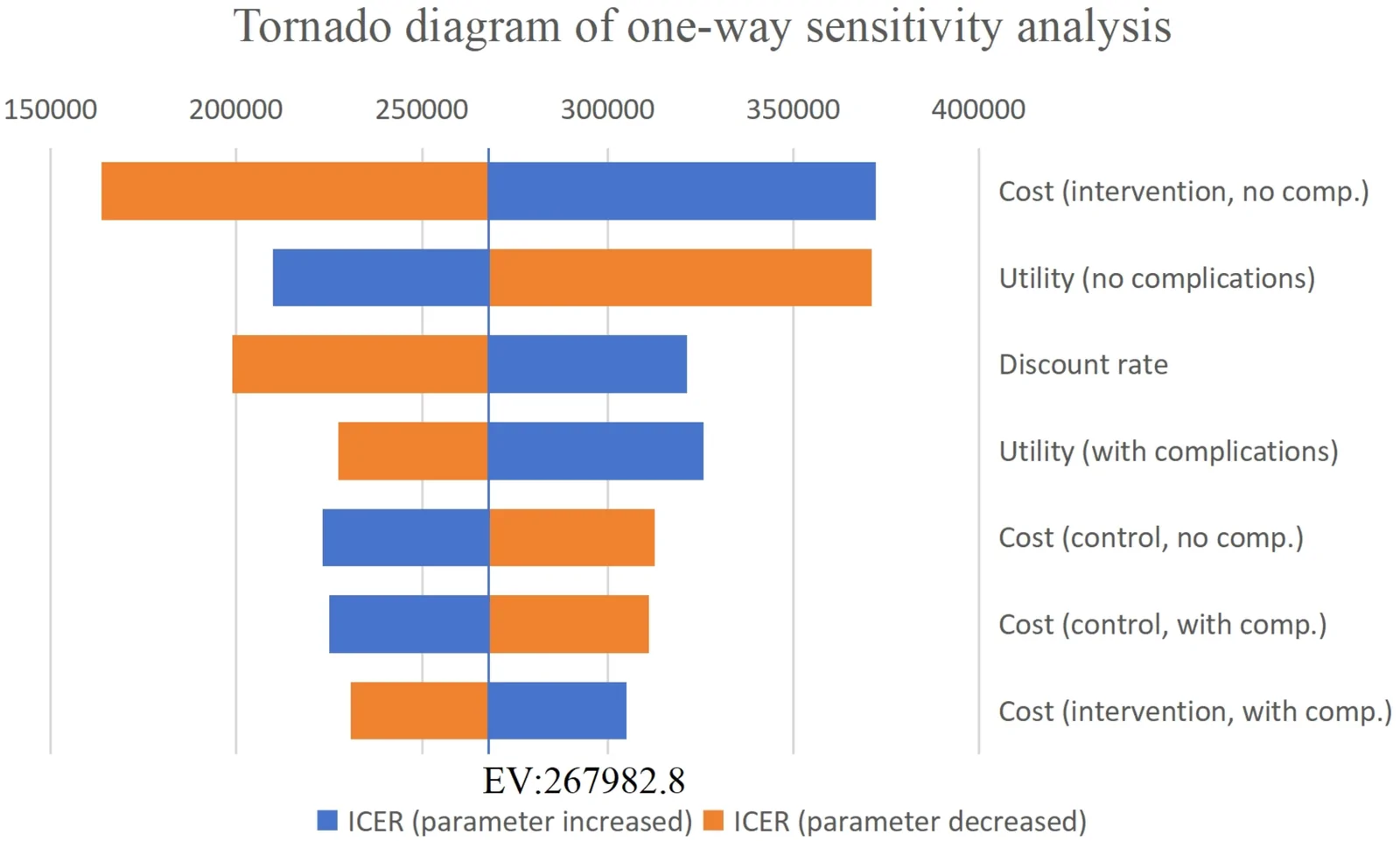
One-way sensitivity analysis tornado diagram.

The results of the one-way sensitivity analysis are illustrated in **Figure 4**. As shown in **Figure 4**, the factor with the greatest influence on the ICER was the cost of the intervention group without complications. Other influencing factors included the health utility value of diabetes without complications, the health utility value of diabetes with complications, the cost of the control group without complications, and the cost of the intervention group with complications.

### 3.3 Results of scenario analysis

When the cost of liraglutide was reduced by 20%, 40%, and 60% based on its original price, the unit price was 252.22 CNY, 189.16 CNY, and 126.11 CNY per pen, respectively, with corresponding annual average costs of 6137.25 CNY, 4602.94 CNY, and 3068.63 CNY, respectively. The cost-utility results after 30 cycles of model simulation are presented in **Table 6**.

**Table 6:** Results of cost-utility analysis after price reduction by liraglutide.

Treatment regimen	Cumulative cost/CNY	Incremental cost/CNY	Cumulative utility/QALYs	Incremental utility/QALYs	ICER/ (CNY/QALY)
Control group	122192.08	—	11.910	—	—
Intervention group (20% price reduction)	173331.21	51139.13	12.188	0.278	183953.71
Intervention group (40% price reduction)	149860.52	27668.44	12.188	0.278	99526.77
Intervention group (60% price reduction)	126389.83	4197.75	12.188	0.278	15099.82

As shown in **Table 6**, the ICER of the intervention group decreased significantly as the price of liraglutide decreased. When the price of liraglutide was reduced by 40%, the ICER was 99526.77 CNY/QALY, which was lower than China’s per capita GDP (99665 CNY). At this point, the additional cost was fully justified, and the regimen exhibited a stronger cost-utility advantage.

## 4 Discussion

The pathogenesis of T2DM is complex and disease progression reduces patients’ quality of life. Moreover, the occurrence of complications resulting from disease progression substantially increases medical expenses for diabetic patients in China (23) and places significant economic pressure on medical insurance payers. Therefore, identifying treatment regimens that meet patients’ therapeutic needs and are cost effective is particularly important. Although previous literature has examined the cost-effectiveness of this drug class, the present study offers differentiated evidence by adopting a Chinese healthcare payer perspective (24).This study constructed a Markov model based on China’s health system to evaluate the long-term costs and utilities of liraglutide versus sitagliptin, each combined with metformin, for the treatment of T2DM. After 30 cycles of simulation, the cohort results showed that compared with the control group, the intervention group had an 8.97% lower transition probability to complications and a 3.77% lower transition probability to death in the treatment of T2DM. A cost–utility analysis revealed that compared with that of the control regimen, the ICER of the intervention regimen for T2DM was 267,985.96 CNY/QALY, which was lower than the WTP threshold of 298,995 CNY/QALY. This finding indicated that the additional cost of liraglutide plus metformin for T2DM was acceptable and conferred a cost-utility advantage. Sensitivity analysis revealed that the probability of the intervention regimen being cost-effective was 63.6%. The three factors with the greatest influence on the ICER were the cost of the intervention group without complications, health utility value of T2DM without complications, and the health utility value of T2DM with complications. Scenario analysis revealed that after the price reduction of liraglutide, the ICER of the intervention regimen for T2DM decreased markedly, leading to an even greater economic advantage. A WTP survey among Chinese diabetes patients found that the median of the mean WTP for complete normalization of HbA1c, fasting blood glucose, and 2-hour postprandial blood glucose was 1000 RMB per month, equivalent to 12,000 RMB per year, which is substantially lower than the ICER value in this study (25).

This study has several limitations: (1) Only direct medical costs were considered, whereas indirect costs and intangible costs were excluded; (2) In the Markov model, transition probabilities were set as fixed values without considering their temporal variations, which may differ from real-world conditions; and (3) Due to the lack of authoritative utility values for Chinese patients with T2DM, the utility data were derived from foreign population studies, which may lead to deviations from values in the Chinese population. (4) We assume 100% adherence tends to overestimate the QALYs gained and total treatment cost. (5) In this study, we aggregated macrovascular and microvascular complications into a single category. We acknowledge that this approach may have underestimated the acute care costs of cardiovascular events and overestimated QALY gains, potentially biasing the ICER.(6) There are notable differences between the DCCT population and the patient cohort enrolled in the present study in terms of baseline characteristics, treatment context, and the level of risk factor control. Therefore, directly extrapolating the glycemic effect observed in DCCT to our study population may introduce bias into the findings.(7) In our model, we assumed that patients remained on their initially assigned treatment throughout the 30-year simulation horizon, without accounting for treatment intensification due to disease progression. In real-world practice, however, patients may require additional therapies as the disease advances, leading to increased long-term costs and potentially biasing our findings. Although the above limitations introduced a certain degree of bias into the study results, the model did not neglect the impact of complications on overall costs and therapeutic outcomes. Thus, this study has a certain practical application value.

In conclusion, compared with sitagliptin plus metformin, liraglutide plus metformin is likely to have a long-term cost-utility advantage at the pre-specified WTP threshold in the treatment of T2DM inadequately controlled by metformin alone.

## Data Availability

The original contributions presented in the study are included in the article/supplementary material. Further inquiries can be directed to the corresponding author.
